# PTEN in prefrontal cortex is essential in regulating depression-like behaviors in mice

**DOI:** 10.1038/s41398-021-01312-y

**Published:** 2021-03-26

**Authors:** Xiao-Qing Wang, Lei Zhang, Zhong-Yuan Xia, Jia-Yin Chen, Yiru Fang, Yu-Qiang Ding

**Affiliations:** 1 Key Laboratory of Arrhythmias, Ministry of Education of China, East Hospital, and Department of Anatomy and Neurobiology, Tongji University School of Medicine, Shanghai, 200092 China; 2 Department of Anatomy, Histology and Embryology, School of Basic Medical Sciences, Fudan University, Shanghai, 200032 China; 3 Shanghai Tenth People’s Hospital, Clinical Medical School of Nanjing Medical University, Nanjing, 211166 China; 4 State Key Laboratory of Medical Neurobiology and MOE Frontiers Center for Brain Science, Institutes of Brain Science, Fudan University, Shanghai, 200032 China; 5 Clinical Research Center and Division of Mood Disorders, Shanghai Mental Health Center, Shanghai Jiao Tong University School of Medicine, Shanghai, 200030 China; 6 CAS Center for Excellence in Brain Science and Intelligence Technology, Shanghai, 200031 China; 7 Department of Laboratory Animal Science, Fudan University, Shanghai, 200032 China

**Keywords:** Depression, Molecular neuroscience

## Abstract

Chronic stress is an environmental risk factor for depression and causes neuronal atrophy in the prefrontal cortex (PFC) and other brain regions. It is still unclear about the molecular mechanism underlying the behavioral alterations and neuronal atrophy induced by chronic stress. We here report that phosphatase and tensin homolog deleted on chromosome ten (PTEN) is a mediator for chronic stress-induced depression-like behaviors and neuronal atrophy in mice. One-month chronic restraint stress (CRS) up-regulated PTEN signaling pathway in the PFC of mice as indicated by increasing levels of PTEN, p-MEK, and p-ERK but decreasing levels of p-AKT. Over-expression of *Pten* in the PFC led to an increase of depression-like behaviors, whereas genetic inactivation or knockdown of *Pten* in the PFC prevented the CRS-induced depression-like behaviors. In addition, systemic administration of PTEN inhibitor was also able to prevent these behaviors. Cellular examination showed that *Pten* over-expression or the CRS treatment resulted in PFC neuron atrophy, and this atrophy was blocked by genetic inactivation of *Pten* or systemic administration of PTEN inhibitor. Furthermore, possible causal link between *Pten* and glucocorticoids was examined. In chronic dexamethasone (Dex, a glucocorticoid agonist) treatment-induced depression model, increased PTEN levels were observed, and depression-like behaviors and PFC neuron atrophy were attenuated by the administration of PTEN inhibitor. Our results indicate that PTEN serves as a key mediator in chronic stress-induced neuron atrophy as well as depression-like behaviors, providing molecular evidence supporting the synaptic plasticity theory of depression.

## Introduction

Depression is a common and devastating illness, which leads to an elevated risk for suicide^1,2^, as well as increased risks of cardiac disease, cerebrovascular disorders, and other medical causes of mortality^3^. Susceptibility to depression is influenced by a variety of genetic, epigenetic, endocrine, and environmental risk factors^4,5^. For instance, vulnerable individuals exposed to traumatic or stressful life events may develop depression^6^, and whether they develop the disease is determined by genetic make-up, exposure to prior stressful experiences, and other physiological parameters^7^. Studies in rodent models have demonstrated that chronic stress causes depression-like behaviors, such as decreased sucrose intake, social defeat, and learned helplessness^8–10^, as well as the atrophy of neuronal processes and decrease of synapse number in the prefrontal cortex (PFC) and other brain regions^2,11,12^. However, it is still unclear for the molecular mechanism underlying the cellular alterations and behavioral changes induced by chronic stress.

PTEN (phosphatase and tensin homolog deleted on chromosome ten) is one of the most frequently lost or mutated tumor suppressors in human cancer, and is generally associated with advanced and metastatic disease^13–15^. PTEN is a negative regulator of the phosphatidylinositol 3-kinase (PI3K)/protein kinase B (AKT)/mammalian target of rapamycin (mTOR) signaling pathway, and is also involved in the brain development, axonal regeneration, and neurodegenerative diseases^16–19^. PTEN deficiency in mouse brain has been shown to lead to behavioral abnormalities, such as altered sociability, repetitive behaviors, and anxiety^20,21^. In addition, the elevated PTEN levels and lowered PI3K and Akt activities have been reported in the brain of depressed suicide victims^22,23^, and over-expression of PTEN causes a reduction of dendrite complexity^24,25^. We thus hypothesized that PTEN may be involved in chronic stress-induced neuronal atrophy and depression-like behaviors.

A hallmark of the stress response is the activation of the hypothalamic–pituitary–adrenal (HPA) axis and increased levels of circulating glucocorticoids, providing maximum physiological support in the acute phase of the ‘fight-or-flight’ response^1,26^. Depression is associated with the hyperactivity of HPA axis^27^, and PTEN expression is up-regulated by the administration of glucocorticoids in A549 cells^28^. Notably, chronic exposure of glucocorticoids causes the atrophy of neurons in the PFC and hippocampus^12,29^, and depression-like behaviors in rodents^30^, raising the possibility that elevated glucocorticoids may contribute to the up-regulation of PTEN in the brain.

In this study, we found that the PTEN levels were increased in the PFC of mice treated with chronic restraint stress (CRS) or chronic administration of dexamethasone (Dex), a synthetic glucocorticoid agonist. Depression-like behaviors were also observed in mice with over-expression of *Pten* in the PFC, while deletion or knockdown of *Pten* in the PFC prevented CRS-induced depression-like behaviors. In addition, systemic administration of PTEN inhibitor reduced the CRS- or Dex-induced depression-like behaviors as well. Neuron atrophy was present in the PFC of CRS- or Dex-treated mice and was blocked by the administration of PTEN inhibitor. These data indicate that PTEN is a key factor in regulating depression-like behaviors in mice, providing a novel and promising strategy of inhibiting PTEN activity for treating this psychiatric disease.

## Materials and methods

### Animals

Eight-week-old adult male mice including wild-type C57BL/6 mice (SLAC Laboratories, Shanghai, China), *Pten*^Flox/Flox^ mice^31^ (#006440; Jackson Laboratory, ME) and heterozygous Emx1-Cre mice^32^ (#005628; Jackson Laboratory) were used. *Pten*^Flox/Flox^ mice were obtained by crossing male and female *Pten*^Flox/Flox^ mice, and heterozygous Emx1-Cre mice were obtained by crossing homozygous Emx1-Cre and wild-type C57BL/6 mice. Mice were housed in groups of 5 in a cage with a 12-h light/dark cycle (lights on at 7:00 a.m.) under controlled temperature (22 ± 2 °C) and humidity (50 ± 10%), and were provided standard diet and water ad libitum. Animal care practices and all experiments were reviewed and approved by the Laboratory Animal Committee of Tongji University School of Medicine, Shanghai, China (TJmed-010-10).

### Intracranial viral injections

Mice were anesthetized with sodium pentobarbital (1 g/kg body weight) and 500 nl of adeno-associated virus (AAV) (1.7 to 1.9 × 10^13^ vg/ml; Taitool Bioscience, Shanghai, China. For details, see below) was injected into the bilateral PFC using pulled glass capillary pipettes according to the atlas (0.5 mm lateral to the midline, 1.54 mm anterior to Bregma, and 1.8 mm below the skull) in a double-blind way. The pipette was left in place for 5 min after each injection and then was slowly withdrawn. Silk sutures were used to close the wound after the injection. For behavioral tests, undiluted viral solution was used, while for neuron morphological analysis, a 1:2000 dilution of virus in phosphate buffer saline (PBS) was injected.

Viruses used in this study included AAV2/8-hSyn-DIO-tdTomato-WPRE-bGHpA (for short, AAV2/8-DIO-tdTomato, which expressed Cre-dependent tdTomato in neurons), AAV2/8-hSyn-DIO-tdTomato-P2A-PTEN-WPRE-pA (AAV2/8-DIO-tdTomato-PTEN, which expressed Cre-dependent tdTomato and PTEN separately in neurons), AAV2/9-hSyn-GFP-3Flag-WPRE-pA (AAV2/9-GFP, which expressed GFP in neurons), AAV2/9-hSyn-Cre-GFP-WPRE-pA (AAV2/9-Cre, which expressed GFP-fused Cre in neurons), AAV2/9-hSyn-DIO-mCherry-miRNA (AAV2/9-DIO-mCherry-miRNA, which expressed Cre-dependent mCherry and scramble miRNA separately in neurons), and AAV2/9-hSyn-DIO-mCherry-PTEN-miRNA (AAV2/9-DIO-mCherry-PTEN-miRNA, which expressed Cre-dependent mCherry and PTEN-miRNA separately in neurons).

The scramble miRNA sequence is 5ʹ-GTCTCCACGCGCAGTACATTT-3ʹ and the PTEN miRNA sequence is 5ʹ-TCGACTTAGACTTGACCTATA-3ʹ.

### Chronic restraint stress (CRS)

Mice were subjected to periodic physical restraint by immobilization in a mouse restraint apparatus (6 h/day)^33,34^ for 30 days, and unrestrained control mice were raised in home cage as usual. The body weight was measured every 5 days. After completion of CRS, mice were subjected to the examination of depression-like behaviors (see below) the next day.

### Chronic Dex treatment

Mice were subcutaneously administered with saline or Dex (0.2 mg (10 ml)/kg body weight; HY-14648, MedChemExpress, NJ) dissolved in saline daily for 21 consecutive days in a double-blind way. The body weight was measured every 5 days, and mice were subjected for examination of depression-like behaviors after the 21-day injection of Dex.

### Drug administration

VO-Ohpic trihydrate (VO-Ohpic), an inhibitor of PTEN^35^ (10 μg (10 ml)/kg body weight; HY-13074, MedChemExpress), was freshly dissolved in sterilized PBS (pH 7.2), and intraperitoneally injected once per day into the mice with 30-day CRS treatment starting on day 15, or into those with 21-day Dex treatment starting on day 11, according to previous reports^36–38^, with modifications. The injection (VO-Ohpic or PBS) was done 1 h before the CRS or Dex treatment in a double-blind way, and the mice were finally subjected to the behavioral observation after the completion of 30-day CRS or 21-day Dex treatment.

### Hormone assays

Mice were fasted for 8–10 h, and blood samples were collected at 8:00 a.m. the next morning. Serum levels of corticosterone (CORT) were measured using Mouse Cortisol ELISA Kit (B163545, BIM Biosciences, CA) according to the manufacturer’s protocol. The concentrations of CORT were then calculated from the appropriate standard curve and expressed as ng/ml.

### Quantitative real-time PCR (RT-PCR)

PFC tissues were isolated and total RNA was extracted using Trizol reagent (RR047A, TaKaRa, Beijing, China) following the manufacturer’s instructions. Three μg of total RNA was subjected to cDNA synthesis. Primers for the CRH (corticotropin-releasing hormone): 5ʹ-CCTCAGCCGGTTCTGATCC-3ʹ (upstream) and 5ʹ- GCGGAAAAAGTTAGCCGCAG-3ʹ (downstream). The primers for GAPDH: 5ʹ- AGGTCGGTGTGAACGGATTTG-3ʹ (upstream) and 5ʹ- TGTAGACCATGTAGTTGAGGTCA-3ʹ (downstream). Real-time PCR was performed using QuantiFast SYBR Green PCR Kit (204156, Qiagen, Shanghai, China), and the reaction solution consisted of 0.4 μl/0.4 μl upstream/downstream primers, 3.2 μl nuclease-free H_2_O, 1 μl cDNA and 5 μl 2x Mix. PCR conditions used were: 10 min at 95 °C for enzyme activation followed by 40 cycles of 15 s denaturation at 95 °C and 1 min anneal/extend at 60 °C. CRH expression levels were normalized to GAPDH with fold change differences determined using the 2^-ΔΔCt^ method.

### Behavioral tests

At the age of 12 weeks, mice were sequentially subjected to the sucrose preference test (SPT), tail suspension test (TST), forced swim test (FST), and open-field test (OFT)^39–41^ in a double-blind way. The SPT was used to test anhedonia-like behavior. Briefly, mice were given a free choice between two bottles of the same color: one with 0.1% sucrose solution and the other with water. It was noteworthy that the position of the bottles was changed after 12 h. At the end of 24 h, liquid consumption was measured, and the sucrose preference was calculated as the ratio of the volume of 0.1% sucrose solution consumed to the total liquid intake. In the TST and FST, the immobility time was recorded in the last 4 min. Locomotor activity was assessed in open field (40 cm × 40 cm) and total distance was measured in 30 min sessions for each animal.

### Analysis of neuronal morphology

Brain slices (35-μm thick) were collected 30 or 21 days after intracranial viral injections. Confocal images of individual pyramidal neurons in layers II/III of PFC were captured with 40X oil objective lens at the resolution of 1024 × 1024 pixels by Nikon A1R laser-scanning confocal microscope. The cell soma size was measured by outlining the soma and calculation of area in squared micrometers. For Sholl analysis, concentric circles with 10 μm differences in radius were drawn around the cell soma, and the number of dendrites intercrossing each circle was counted. The neuronal dendrites were traced and total dendritic length was calculated^25,42^. All the morphological analysis was done in a double-blind way.

### Immunofluorescence

Brain slices (35-μm thick) were pretreated with sodium citrate (0.05 M, pH 6.0) for 5 min at 95 °C for antigen retrieval and this procedure might largely diminish the GFP or tdTomato fluorescence. Then these slices were incubated with rabbit anti-PTEN antibody (1:1000; ab32199, Abcam, Shanghai, China), anti-p-AKT antibody (1:1000; #4060, CST, MA), or goat anti-GFP antibody (1:2000; NB100-1770, Novus Biologicals, CO) at 4 °C overnight, and incubated with biotinylated horse anti-rabbit IgG (1:500; Jackson ImmunoResearch, PA) or Alexa Fluor 488 donkey anti-goat IgG (1:500; Invitrogen, Shanghai, China) at room temperature for 3 h, followed by incubation with streptavidin-Cy3 or streptavidin-Cy5 (1:1000; Jackson ImmunoResearch) and counterstaining with Hoechst 33258 (1:2000, Sigma, Shanghai, China) at room temperature for 10 min. Images were captured with a Nikon A1R laser-scanning confocal microscope.

### Western blots

The PFC tissue was dissected out and lysed with cold RIPA lysis buffer (#89901, Invitrogen). The collected protein was diluted in sample buffer (LT101s, EpiZyme, MA) and boiled for 10 min. Twenty μg of protein was loaded onto 10% SDS-PAGE gel and transferred to nitrocellulose membranes. Antibodies used for Western blot were rabbit anti-PTEN (1:1000; ab32199, Abcam), rabbit anti-p-PTEN (1:1000; #9551, Abcam), mouse anti-p-ERK1/2 (1:1000; #4370, CST), mouse anti-ERK1/2 (1:1000; #4696, CST), rabbit anti-p-MEK1 (1:1000; #26975, CST), rabbit anti-MEK1 (1:1000; #12671, CST), rabbit anti-AKT (1:1000; #4691, CST), rabbit anti-p-AKT(1:1000; #4060, CST), and rabbit anti-GAPDH (1:2000; LF206, EpiZyme). Protein bands were detected using enhanced chemiluminescence reagents (#1525703, Millipore, Hong Kong, China).

### Statistical analysis

All data were tested for normal distribution and statistical analysis was then carried out using two-tailed Student’s *t*-test and one-way or two-way ANOVA with post hoc Tukey’s test (GraphPad Prism, v8.0). Data are presented as mean ± SEM. *p* values less than 0.05 were considered significant. All experiments were replicated for at least 3 times.

## Results

### CRS treatment up-regulates PTEN levels in the PFC

**Fig. 1:**
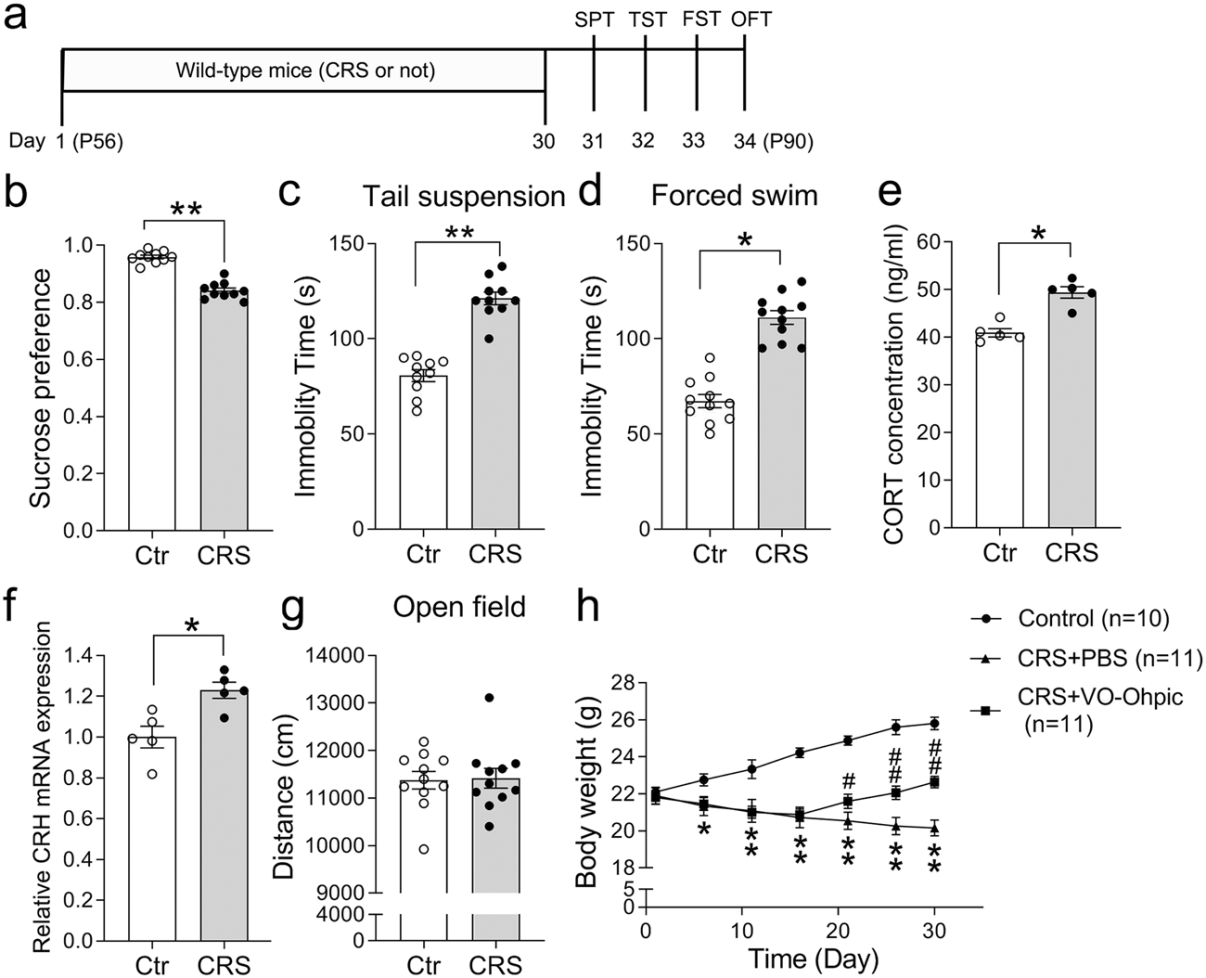
CRS treatment induces depression-like behaviors and elevates HPA activity. **a** Diagram of experiment design and timeline. **b**–**d** The CRS treatment resulted in decreased sucrose preference (**b**) and increased immobility time in the TST (**c**), and FST (**d**). *n* = 10 for each group in sucrose preference test and TST, *n* = 11 for each group in FST. **e**, **f** The CRS treatment significantly increased the serum levels of CORT (**e**) and transcription levels of CRH in the PFC (**f**). **g** There were no significant changes in the total traveled distance in the OFT. *n* = 11 for each group. **h** A significant difference was observed in the body weight between CRS-treated mice and control mice during the 30-day period, but the reduction of body weight in CRS-treated mice could be partially reversed when PTEN inhibitor VO-Ohpic was intraperitoneally injected once per day from day 15. All the data are presented as mean ± SEM. Data were analyzed using Student’s *t*-tests (**b**–**g**) and two-way repeated measures ANOVA (**h**). *p < 0. 05, **p < 0.01 (control mice versus CRS-treated mice); ^#^*p* < 0.05, ^##^*p* < 0.01 (CRS-treated mice versus CRS-treated mice with VO-Ohpic). CRS, chronic restraint stress; FST, forced swim test; OFT, open-field test; SPT, sucrose preference test; TST, tail suspension test; VO-Ohpic, VO-Ohpic trihydrate.

CRS is widely used in inducing depression-like behaviors in rodents^43,44^. To validate our CRS protocol, the behavior tests including the SPT, TST, FST, and OFT were performed (Fig. 1a). As expected, CRS treatment resulted in a decrease of sucrose preference and an increase of immobility time in both TST and FST (Fig. 1b–d). Because the hyperactivity of the HPA axis is involved in the pathogenesis of depression^45,46^, CRS-treated mice exhibited increased CORT levels in the serum, and increased CRH transcription in the PFC (Fig. 1e, f), consistent with the previous results^47^. The locomotor activity was not changed in CRS-treated mice as shown by similar total traveled distance relative to controls (Fig. 1g). Finally, there was a gradual increase of body weight in control mice during the 30-day period, but this was not present in CRS-treated mice (Fig. 1h). Taken together, our CRS treatment induced the appearance of depression-like behaviors in mice.

**Fig. 2:**
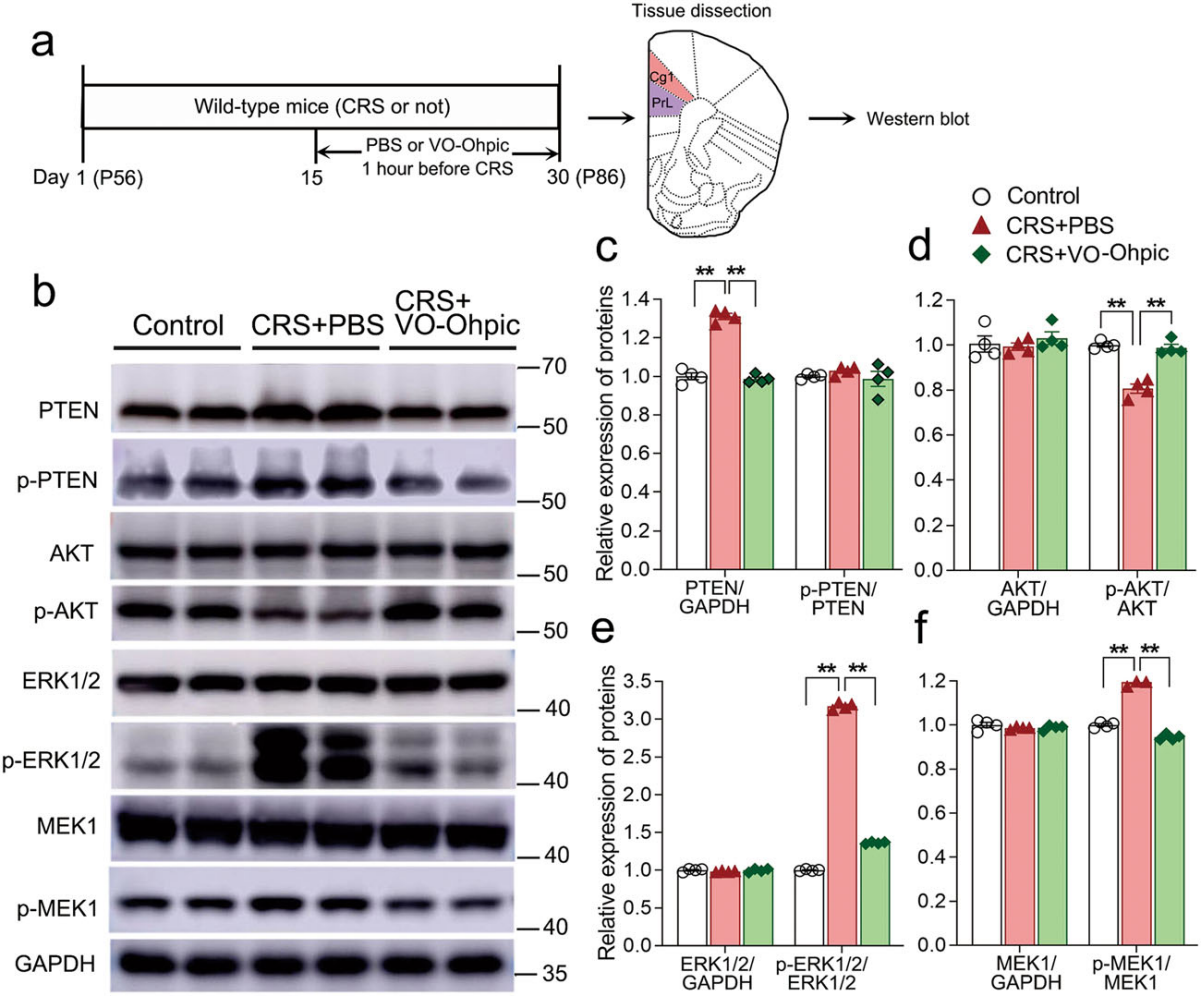
The activity of PTEN signaling pathway is up-regulated in the PFC of CRS-treated mice, and it can be prevented by the administration of PTEN inhibitor. **a** Diagram of experiment design and timeline. Cortical tissues from the two subregions of PFC, Cg1, and PrL were collected for Western blots. **b**–**f** One-month CRS treatment led to the increase in the levels of PTEN, p-MEK1, and p-ERK1/2 and decrease of p-AKT in the PFC, but these changes were not observed in the CRS-treated mice receiving daily injection of PTEN inhibitor during the period of days 15–30. Note that the levels of p-PTEN, ERK1/2, AKT, and MEK1 were not changed by the CRS-treatment or application of PTEN inhibitor. *n* = 4 in each group and data are presented as mean ± SEM. Two-way ANOVA with post hoc Tukey’s test, *p < 0.05, **p < 0.01. Cg1, cingulate cortex, area 1; CRS, chronic restraint stress; PrL, prelimbic cortex; VO-Ohpic, VO-Ohpic trihydrate.

To explore if PTEN is involved in CRS-induced depression, we examined the expression levels of PTEN and its associated molecules in the PFC (Fig. 2a), which is a critical brain region for depression^48,49^. Western blots showed that the levels of PTEN were significantly increased in CRS-treated mice (Fig. 2b, c). The components of PTEN signaling pathway showed corresponding changes as reflected by the increased phosphorylation of ERK1/2 and MEK1, and decreased phosphorylation of AKT in CRS-treated mice (p-ERK1/2/ERK1/2, p-MEK1/MEK1, and p-AKT/AKT ratio; Fig. 2b, d–f). Note that the levels of PTEN phosphorylation, AKT, ERK1/2 and MEK1 were not changed (Fig. 2b–f). Thus, our data demonstrate that PTEN signaling pathway is up-regulated in CRS-treated mice, suggesting possible involvement of PTEN in the development of depression-like behaviors in mice.

### PTEN in the PFC is critical for depression-like behaviors in mice

**Fig. 3:**
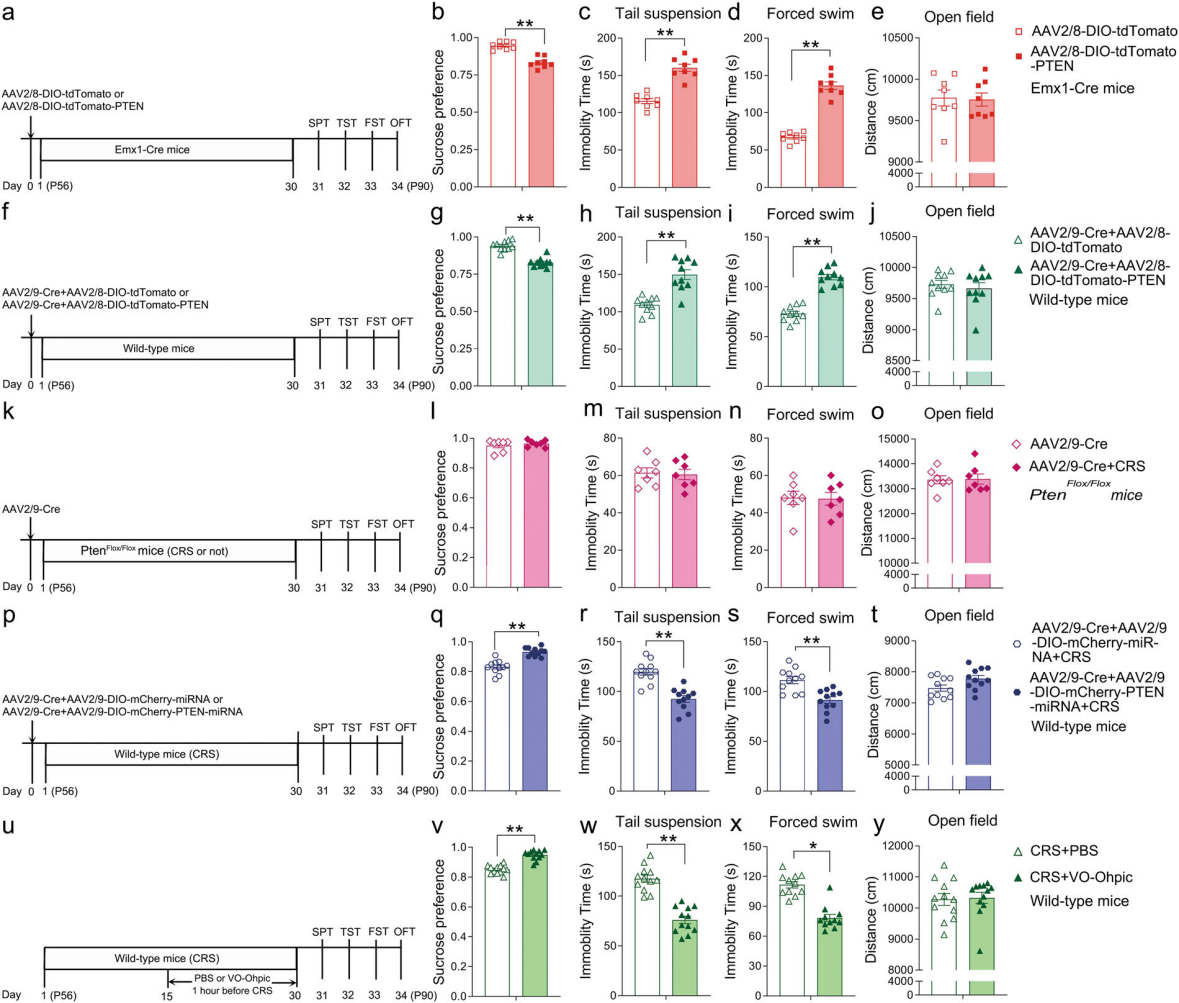
PTEN in the PFC is essential in the regulation of depression-like behaviors in mice. **a** Diagram of experiment design and timeline for (**b**–**e**). **b**–**e** Compared with control mice, the mice with over-expression of PTEN in the PFC showed decreased sucrose preference (**b**), increased immobility time in the TST (**c**), and FST (**d**), no changes in the total traveled distance in OFT (**e**). Cre-dependent over-expression of *Pten* was achieved in Emx1-Cre mice. *n* = 8 in each group. **f** Diagram of experiment design and timeline for (**g–j**). **g**–**j** Wild-type mice with over-expression of PTEN in the PFC showed decreased sucrose preference (**g**) and increased immobility time in the TST (**h**) and FST (**i**) without obvious changes in the total traveled distance in the OFT (**j**). *n* = 10 in each group. **k** Diagram of experiment design and timeline for (**l**–**o**). **l**–**o** Genetic inactivation of *Pten* in the PFC of *Pten*^Flox/Flox^ mice with one-month CRS showed no differences in the sucrose preference (**l**), immobility time in the TST (**m**), and FST (**n**), or total distance traveled in the OFT (**o**) compared with control *Pten*^Flox/Flox^ mice raised in home cage. *n* = 7 in each group. **p** Diagram of experiment design and timeline for (**q**–**t**). **q**–**s** The decreased sucrose preferences (**q**) and increased immobility time in the TST (**r**), and FST (**s**) in one-month CRS-treated wild-type mice were significantly attenuated with knockdown of *Pten* in the PFC. **t** Knockdown of *Pten* did not affect the traveled distance shown by the OFT. *n* = 11 in each group. **u** Diagram of experiment design and timeline for (**v**–**y**). **v**–**x** The sucrose preference is improved (**v**), and the immobility time in TST (**w**), and FST (**x**) is reduced in CRS-treated mice by VO-Ohpic treatment. **y** The locomotor activity shown by OFT is not significantly changed in CRS-treated mice with the application of VO-Ohpic. *n* = 12 in each group. All data are presented as mean ± SEM. Student’s *t*-tests, *p < 0.05, **p < 0.01. CRS, chronic restraint stress; FST, forced swim test; OFT, open-field test; SPT, sucrose preference test; TST, tail suspension test; VO-Ohpic, VO-Ohpic trihydrate.

To explore possible effects of increased PTEN in the PFC on depression-like behaviors, we injected Cre-dependent PTEN-overexpressing AAV (AAV2/8-DIO-tdTomato-PTEN) or control virus (AAV2/8-DIO-tdTomato) into the PFC of Emx1-Cre mice (Fig. 3a). Injection of AAV2/8-DIO-tdTomato-PTEN resulted in an increase of PTEN levels in Emx1-Cre mice compared to those with injection of control AAV2/8-DIO-tdTomato (Supplementary Fig. 1a). Thirty days later, we performed behavioral examination of Emx1-Cre mice with the injection of virus. Results showed that the sucrose preference was significantly reduced (Fig. 3b) and the immobility time in the TST and FST was obviously increased (Fig. 3c, d) in the mice with injection of AAV2/8-DIO-tdTomato-PTEN compared to those with injection of control AAV2/8-DIO-tdTomato. Note that the total traveled distance in the OFT was not different between the groups (Fig. 3e), while the entries to the center region were decreased in AAV2/8-DIO-tdTomato-PTEN-injected Emx1-Cre mice (Supplementary Fig. 2). Similarly, we performed over-expression of PTEN by co-injection of AAV2/9-Cre with AAV2/8-DIO-tdTomato-PTEN into the PFC of wild-type mice (Fig. 3f). There was a marked increase in PTEN immunoreactivity in the cortical neurons co-infected with AAV2/9-Cre and AAV2/8-DIO-tdTomato-PTEN compared to those with AAV2/9-Cre and AAV2/8-DIO-tdTomato (Supplementary Fig. 3). Behavioral examination was performed 30 days after the injection and similar alterations were obtained as mentioned above (Fig. 3g–j). Thus, depression-like behaviors are increased in mice with over-expression of PTEN in the PFC.

Next, we set out to explore if inactivation of *Pten* in the PFC affects depression-like behaviors. In the first set of experiments, we injected AAV2/9-Cre into the PFC of *Pten*^Flox/Flox^ mice to observe how depression-like behaviors are changed in these mice with or without one-month CRS treatment (Fig. 3k). Western blots showed a significant reduction of PTEN levels in the PFC of *Pten*^Flox/Flox^ mice with injection of AAV2/9-Cre compared to those with injection of control AAV2/9-GFP (Supplementary Fig. 1b). At cellular level, the deletion of *Pten* in Cre-expressing neurons was confirmed by immunostaining of PTEN and the presence of intense p-AKT immunoreactivity (Supplementary Fig. 4). Behavioral examination showed that there were no differences in the sucrose preference (Fig. 3l), the immobility time in the TST and FST (Fig. 3m, n), or the total distance traveled in the OFT (Fig. 3o) between the CRS-treated and non-CRS-treated *Pten*^Flox/Flox^ mice with injection of AAV2/9-Cre. Thus, the CRS-induced depression-like behaviors are no longer present in the mice with genetic inactivation of *Pten* in the PFC.

To further confirm this, we performed another set of experiments in wild-type mice with knockdown of *Pten* in the PFC. A mix of Cre-dependent miRNA against *Pten* expression virus (AAV2/9-DIO-mCherry-PTEN-miRNA) and AAV2/9-Cre was injected into the PFC of wild-type mice (Fig. 3p), and the knockdown efficacy was assessed by Western blot (Supplementary Fig. 5). One day after the virus injection, all mice were subjected to the CRS, and behavioral observation was done 30 days later (Fig. 3p). In comparison with control mice with injection of both AAV2/9-Cre and AAV2/9-DIO-mCherry-miRNA, those with injection of AAV2/9-Cre and AAV2/9-DIO-mCherry-PTEN-miRNA exhibited improved sucrose preferences (Fig. 3q) and decreased immobility time in the TST and FST (Fig. 3r, s) with no obvious differences in the total distance traveled in the OFT (Fig. 3t). Taken together, our data obtained by the manipulation of PTEN levels in the PFC demonstrate that PTEN is essential in the regulation of depression-like behaviors in mice.

### PTEN is implicated in CRS-induced atrophy of cortical neurons in mice

**Fig. 4:**
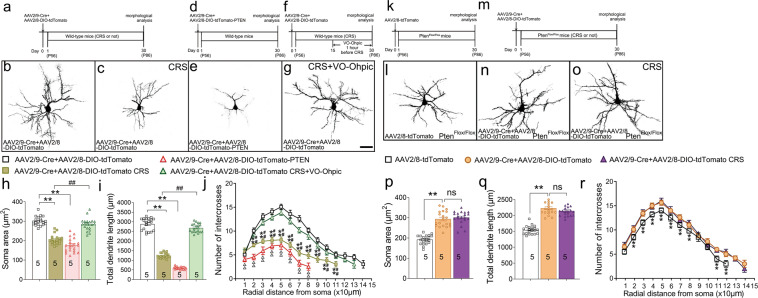
PTEN is a key factor involved in the CRS-induced dendrite atrophy. **a** Diagram of experiment design and timeline for (**b**, **c**). **b**, **c** Representative images showing the dendrite tree of pyramidal neurons in layers II/III of PFC from control mice and CRS-treated mice. **d** Diagram of experiment design and timeline for (**e**). **e** Representative image showing the dendrite tree of pyramidal neurons in layers II/III of PFC from mice with over-expression of *Pten*. **f** Diagram of experiment design and timeline for (**g**). **g** Representative image showing the dendrite tree of pyramidal neurons in layers II/III of PFC from CRS-treated mice with VO-Ohpic administration. **h**–**j** Statistical data showing the soma size (**h**), total length of dendrites (**i**), and dendritic branching (**j**) of the PFC neurons in indicated groups. *n* = 20 cells from 5 mice in each group and all data are presented as mean ± SEM. One-way ANOVA (**h**, **i**), **p < 0. 01 versus controls, ^##^*p* < 0.01 (CRS-treated mice versus CRS-treated mice with VO-Ohpic). Two-way ANOVA with post hoc Tukey’s test (**j**), *p < 0. 05, **p < 0.01 (control mice versus CRS-treated mice); ^#^*p* < 0.05, ^##^*p* < 0.01 (CRS-treated mice versus CRS-treated mice with VO-Ohpic); ^Δ^*p* < 0. 05; ^ΔΔ^*p* < 0. 01 (control mice versus mice with over-expression of *Pten*). **k** Diagram of experiment design and timeline for (**l**). **l** Representative image showing the dendrite tree of pyramidal neuron in layers II/III of PFC from control mice. **m** Diagram of experiment design and timeline for (**n**, **o**). **n**, **o** Representative images showing the dendrite tree of pyramidal neuron in layers II/III of PFC from *Pten*-deficient neurons without or with CRS, respectively. Deletion of *Pten* was achieved by the injection of AAV2/9-Cre in *Pten*^Flox/Flox^ mice. **p**–**r** Statistical data showing the soma size (**p**), total length of dendrites (**q**), and dendritic branching (**r**) of the PFC neurons in indicated groups. *n* = 20 cells from 5 mice in each group and all data are presented as mean ± SEM. One-way ANOVA (**p**, **q**), **p < 0. 01 versus controls. Two-way ANOVA with post hoc Tukey’s test (**r**), *p < 0. 05, **p < 0.01 (control neuron versus *Pten*-deficient neurons with CRS). Scale bar = 40 μm (**b**, **c**, **e**, **g**, **l**, **n**, and **o**). CRS, chronic restraint stress; VO-Ohpic, VO-Ohpic trihydrate.

It has been reported that chronic stress leads to the reduction of dendritic length and spine density of cortical neurons^1,2,12^. Considering the role of PTEN in regulating the dendrite morphology^24^, we speculated that PTEN is involved in the CRS-induced dendrite atrophy which may be one of the cellular mechanisms underlying the onset of depression-like behaviors. To this end, we first examined the dendrite morphology of cortical pyramidal neuron in the layers II/III of PFC of one-month CRS-treated wild-type mice by co-injection of AAV2/9-Cre and AAV2/8-DIO-tdTomato one day before the commencement of the CRS treatment (Fig. 4a); tdTomato was used to trace the dendrite morphology of infected neurons. Consistent with previous reports^50,51^, the atrophy of cortical neurons was evident in the CRS-treated mice (Fig. 4b, c), as shown by significant reductions in the soma size (Fig. 4h), the total length of dendrites (Fig. 4i), and number of dendrite branches (Fig. 4j) relative to those of control mice. Next, we moved to examine how the dendrite tree was changed after the over-expression of *Pten* in the PFC. As mentioned above, the AAV2/9-Cre-GFP and AAV2/8-DIO-tdTomato-PTEN virus was co-injected into the PFC of wild-type mice (Fig. 4d), and tdTomato could depict the dendrite morphology of infected neurons (Fig. 4e). Importantly, the cortical neurons with over-expression of PTEN showed simplified dendrites as indicated by a significant reduction in the total length of dendrites and number of dendrite branches as compared with those of control mice with co-injection of AAV2/9-Cre and AAV2/8-DIO-tdTomato virus (Fig. 4h–j). Thus, the CRS treatment or over-expression of *Pten* leads to the dendrite atrophy of cortical neurons in the PFC.

We then moved to examine the dendrite morphology when *Pten* is inactivated. As described above, a mix of AAV2/9-Cre and AAV2/8-DIO-tdTomato (Fig. 4m) or AAV2/8-tdTomato alone (Fig. 4k) was injected into the PFC of *Pten*^Flox/Flox^ mice. One day after the injection, the mice receiving AAV2/9-Cre and AAV2/8-DIO-tdTomato were randomly divided into two groups: one is raised in home cage for one month, and the other is subjected to one-month CRS treatment (Fig. 4m). Deletion of *Pten* was confirmed by the loss of PTEN immunoreactivity (Supplementary Fig. 4a–c) and increased p-AKT immunoreactivity (Supplementary Fig. 4d–f) in tdTomato-positive neurons compared with adjacent uninfected neurons. Sholl analysis showed that the cortical neurons lacking *Pten* had larger soma size (Fig. 4l, n, p) and increased dendrite length (Fig. 4l, n, q) and branches (Fig. 4l, n, r) compared with control mice. Critically, these alterations of *Pten*-deficient neurons were not observed in the mice with one-month CRS treatment (Fig. 4o–r). These results suggest that the inactivation of *Pten* in PFC neurons endows them with the ability against the CRS-induced neuronal atrophy, which may serve as one of the cellular mechanisms underlying the essential role of PTEN in regulating depression-like behaviors in mice.

### PTEN inhibitor VO-Ohpic prevents the onset of CRS-induced depression-like behaviors in mice

To provide more evidence supporting the critical role of PTEN in regulating depression-like behaviors, we applied VO-Ohpic, an inhibitor of PTEN^35^ by intraperitoneal injection in wild-type mice. VO-Ohpic was applied in one-month CRS-treated mice starting on day 15 until the end of CRS treatment (Figs. 2a, 3u, and 4f). Western blots showed that the 2-week application of VO-Ohpic indeed increased the levels of p-AKT with unchanged total AKT compared to those with CRS treatment only (Fig. 2b, d). In addition, the level of PTEN and phosphorylation of ERK1/2 and MEK1 were reduced in the CRS-treated mice with the injection of VO-Ohpic compared with CRS-treated mice without VO-Ohpic treatment (Fig. 2b, c, e, f), showing that the PTEN activity is suppressed by the application of VO-Ohpic. Behavioral observations (Fig. 3u) revealed that the sucrose preference is improved (Fig. 3v), and immobility time in both TST and FST was reduced compared with controls (Fig. 3w, x). In addition, VO-Ohpic treatment prevented the decrease of body weight in CRS-treated mice (Fig. 1h) without affecting locomotor activity shown by OFT (Fig. 3y). Lastly, inhibition of PTEN with VO-Ohpic prevented the atrophy induced by CRS (Fig. 4g–j). Thus, systemic administration of VO-Ohpic is able to prevent the development of CRS-induced depression-like behaviors in mice.

### Elevated glucocorticoids are involved in PTEN-regulated depression-like behaviors in mice

Our results showed the increase of serum CORT levels and CRH transcriptions in the PFC of the CRS-treated mice, and increased activity of HPA is believed to be one of the mechanisms underlying the development of depression^52^. It is likely that the elevated glucocorticoids contribute to the increased PTEN activity and the depression-like behaviors in the CRS-treated mice. Dex is a synthetic glucocorticoid agonist and chronic Dex treatment is widely used for inducing depression-like behaviors in rodents^53–55^, and thus it was used to explore: (i) how the PTEN levels are changed in Dex-treated mice, and (ii) whether suppressing PTEN activity is able to attenuate the depression-like behaviors in Dex-treated mice.

**Fig. 5:**
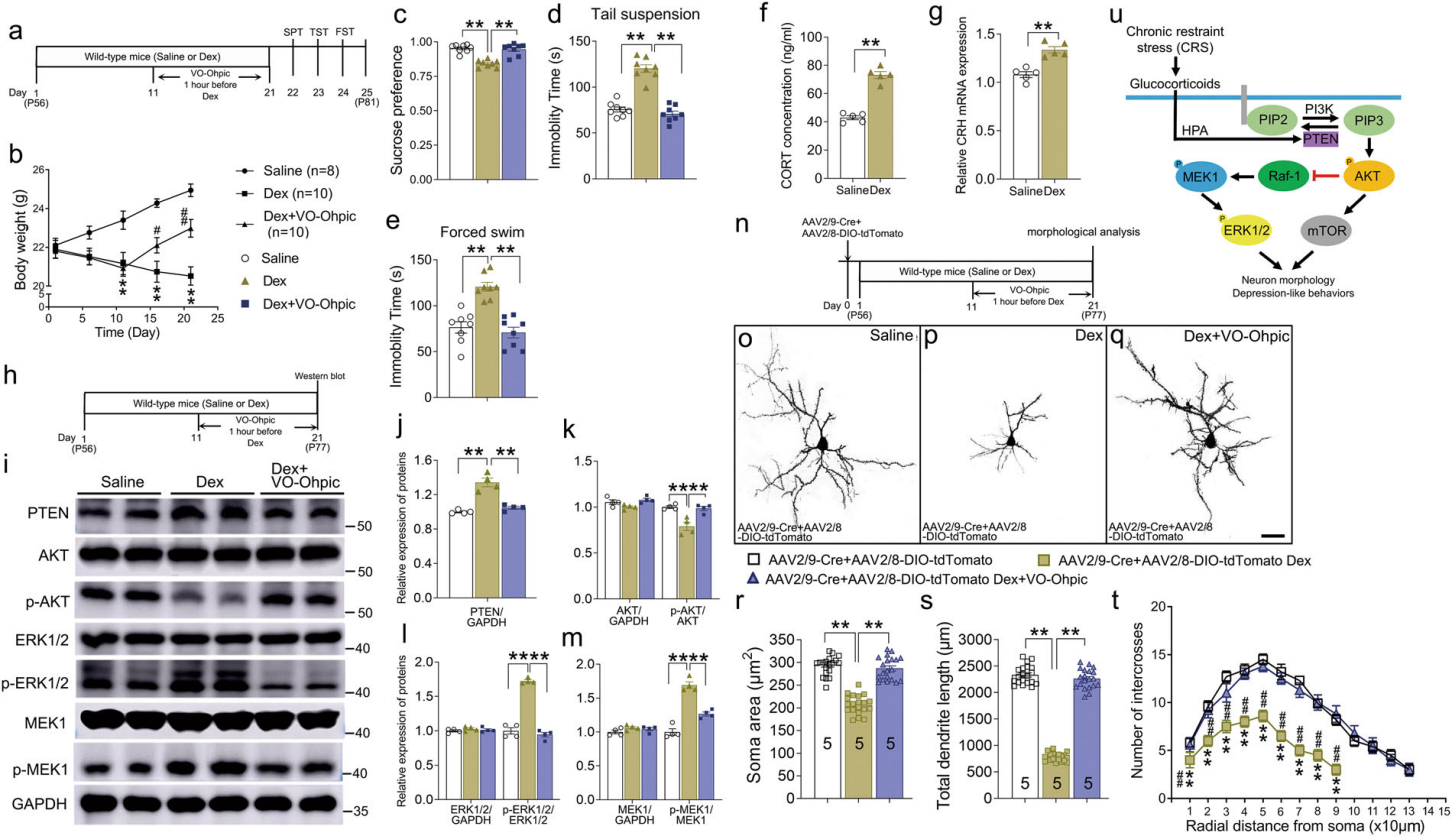
PTEN inhibitor VO-Ohpic prevents depression-like behaviors and neuron atrophy in Dex-treated mice. **a** Diagram of experiment design and timeline for (**b**–**e**). **b** A significant reduction in body weight was observed after 21-day Dex treatment, but it could be partially reversed by intraperitoneal injection of VO-Ohpic into the mice starting on day 11. Data were analyzed using two-way repeated measures ANOVA, *p < 0. 05, **p < 0.01 (Saline versus Dex-treated mice); ^#^*p* < 0.05, ^##^*p* < 0.01 (Dex-treated mice versus Dex-treated mice with VO-Ohpic). **c**–**e** Dex resulted in decreased sucrose preference (**c**), and increased immobility time in TST (**d**) and FST (**e**). *n* = 8 in each group and data were analyzed using one-way ANOVA, **p < 0.01. **f**, **g** The levels of CORT in the serum and CRH transcription in the PFC were increased in Dex-treated mice. Data were analyzed using Student’s *t*-tests, **p < 0.01. **h** Diagram of experiment design and timeline for (**i**–**m**). **i**–**m** Three-week Dex treatment led to increased levels of PTEN, p-MEK1, and p-ERK1/2 and reduced level of p-AKT, and these changes were reversed by VO-Ohpic starting on day 11. Note that the levels of ERK1/2, AKT, and MEK1 were not changed by the Dex treatment or application of PTEN inhibitor. *n* = 4 in each group and data are presented as mean ± SEM. One-way ANOVA (**j**), *p < 0.05, **p < 0.01. Two-way ANOVA with post hoc Tukey’s test (**k**–**m**), *p < 0.05, **p < 0.01. **n** Diagram of experiment design and timeline for (**o**–**q**). **o**–**q** Representative images of pyramidal neuron in layers II/III of PFC neurons of control mice and Dex-treated mice with or without VO-Ohpic administration, respectively. Scale bar = 40 μm. **r**–**t** Statistical data showing the soma size (**r**), total length of dendrites (**s**), and dendritic branching (**t**) of the PFC neurons in indicated groups. *n* = 20 cells from 5 mice in each group, and all data are presented as mean ± SEM. One-way ANOVA (**r**, **s**), **p < 0.01. Two-way ANOVA with post hoc Tukey’s test (**t**), *p < 0. 05, **p < 0.01 (control mice versus Dex-treated mice); ^#^*p* < 0.05, ^##^*p* < 0.01 (Dex-treated mice versus Dex-treated mice with VO-Ohpic). **u** Model of PTEN signaling pathway in CRS-induced neuronal atrophy and depression-like behaviors. CORT, corticosterone; CRH, corticotropin-releasing hormone; CRS, chronic restraint stress; Dex, dexamethasone; FST, forced swim test; HPA, hypothalamic–pituitary–adrenal axis; OFT, open-field test; SPT, sucrose preference test; TST, tail suspension test; VO-Ohpic, VO-Ohpic trihydrate.

To ensure the validity of the chronic Dex model, the behavior tests including the SPT, TST, and FST were performed (Fig. 5a). As expected, 21-day Dex treatment prevented the increase of body weight (Fig. 5b), and resulted in a decrease of sucrose preference (Fig. 5c) and increase of immobility time in both TST and FST (Fig. 5d, e). Like the CRS-treated mice, serum CORT levels and CRH transcription in the PFC were increased (Fig. 5f, g). Thus, our Dex protocol indeed induced depression-like behaviors in mice. To explore if PTEN and its associated proteins were involved in the Dex-induced depression, we examined the expression levels of PTEN, AKT, p-AKT, ERK1/2, p-ERK1/2, MEK1, and p-MEK1 in the PFC (Fig. 5h). Western blots showed that the levels of PTEN and the phosphorylation of ERK1/2 and MEK1 were significantly increased (Fig. 5i, j, l, m), while the phosphorylation of AKT was decreased in Dex-treated mice (Fig. 5i, k). Thus, our data demonstrate that PTEN activity is up-regulated in the PFC of Dex-treated mice.

Then, the PTEN inhibitor was administrated daily starting on day 11 till the end of Dex treatment on day 21 (Fig. 5a, h). The behavior tests showed that the VO-Ohpic treatment prevented the decrease of body weight (Fig. 5b), improved the sucrose preference, and reduced the immobility time in the TST and FST (Fig. 5c–e). Changes in the levels of PTEN, p-AKT, p-ERK1/2, and p-MEK1 were reversed by VO-Ohpic in Dex-treated mice (Fig. 5i–m). Next, the dendrite morphology of PFC neurons was examined in the same way (Fig. 5n). As reported previously^56^, the 21-day Dex treatment led to PFC neuron atrophy (Fig. 5o, p), revealed by significant reduction of soma size (Fig. 5r), total dendritic length (Fig. 5s), and number of branches (Fig. 5t) relative to those of controls, and these alterations no longer existed after the administration of PTEN inhibitor (Fig. 5o–t). Our data suggest that that glucocorticoids may serve as a mediator in the CRS-induced up-regulation of PTEN and subsequently depression-like behaviors in mice.

## Discussion

In this study, we addressed the role of PTEN in stress-induced depression-like behaviors in mice. First, CRS and Dex treatments up-regulates PTEN levels in the PFC. Second, over-expressing *Pten* in the PFC results in deleterious effects on dendrite tree and the increase of depression-like behaviors, whereas deletion or knockdown of *Pten* is able to prevent CRS-induced depression-like behaviors and neuronal atrophy in the PFC. Thus, a model of PTEN signaling pathway in neuronal morphology and depression-like behaviors is proposed (Fig. 5u). Although the FST is also considered as a measurement for stress response, not directly related to depression^57^, PTEN over-expression in the PFC increased such stress (Fig. 3d, i), which would promote the development of depression. Potential application in treating depression is shown by systemic administration of PTEN inhibitor based on the behavioral and neuronal morphological data. In addition, our data are essentially consistent with another study concerning PTEN in 5-HT neurons in dorsal raphe nucleus (DRN)^38^: (1) PTEN is elevated in 5-HT neurons by CRS; (2) depression-like behaviors are reduced in Pten cKO mice; and (3) dendritic complexity is increased in Pten-deficient 5-HT neurons. Together, these results suggest that PTEN in PFC-DRN network may be critical in regulating depression-like behaviors.

A wealth of evidence indicates that the PFC is a key component of the corticostriatal circuits which are thought to generate pathological emotional behavior and accompanied physiological disturbance^58,59^. Glucocorticoids regulate the termination of the stress response through negative feedback at the level of the hypothalamus and pituitary, as well as other brain regions such as the PFC, hippocampus, and amygdala^60^. A well-documented consequence of chronic stress exposure is impaired negative feedback of the HPA axis with increased levels of corticosteroids and CRH^61–63^, which is observed in the majority of depressed patients^64^ and CRS-treated mice as shown in the present study. The previous studies have observed an increase in PTEN protein levels in the PFC of depressed suicide victims^23^. What could be the remote cause of the PTEN dysregulation in the PFC of depressive brain? It has been reported that PTEN expression is up-regulated by the administration of glucocorticoids in A549 cells^28^. Our data indicate that chronic Dex treatment up-regulates PTEN levels in the PFC, and the application of PTEN inhibitor prevents the onset of Dex-induced depression-like behaviors in mice. On the basis of these findings, it is likely that glucocorticoids serve as a mediator in the stress-induced up-regulation of PTEN and depression-like behaviors in mice.

PTEN mutations were associated with autism spectrum disorder (ASD)^65,66^ and found in about 2% of total ASD patients^67^. A current model suggests PTEN mutation-induced increase of dendritic branching in PFC may disrupt the brain connectivity and then cause ASD-related neurobehavioral deficits^68^. We showed that knockout of PTEN in individual neurons led to hypertrophy (Fig. 4l, n–r) and over-expression of PTEN resulted in neuronal atrophy (Fig. 4c, e, h–j), which supports this model. PTEN is widely expressed in the brain and is present at both pre- and post-synaptic sites^69,70^. The PTEN/PI3K pathway is essential for some important aspects of synaptic function and plasticity. Interestingly, the blockade of Akt-mTOR signaling completely disrupted ketamine-induced synaptogenesis and behavioral responses in animal models of depression^71–75^. Inactivation of PTEN in the differentiated neurons of the cerebral cortex resulted in increased response to sensory stimuli with neuronal hypertrophy, including hypertrophic and ectopic dendrites and axon tracts with increased synapses in mice^76^. In contrast, excess of PTEN restricts neuronal growth, leading to a substantial decrease in dendritic branching and synapse number^77^. In addition, the hippocampal granule neurons or dorsal raphe 5-HT neurons with PTEN deletion receive more synaptic inputs^38,78–80^ and show increased excitability in adult mice^78–80^, and further studies are needed to examine if similar alterations occur in *Pten*-deficient pyramidal neurons in the PFC.

According to the synaptic theory of depression, extrinsic factors such as chronic stress leads to impairments of synaptic plasticity at both morphological and functional levels, and traditional antidepressants (e.g., selective serotonin reuptake inhibitors) and new category of antidepressants (e.g., ketamine) are able to restore the synaptic plasticity via multiple intracellular pathways including AKT-mTOR^1,2,81^. Our results demonstrated essential roles of PTEN, negative regulator of AKT/mTOR signaling, in regulating depression-like behaviors as well as CRS- and Dex-induced neuronal atrophy in mice. Thus, exploring potential chemicals which negatively regulate AKT/mTOR signaling may be helpful for developing new antidepressant. In addition to influencing the AKT-mTOR signaling pathway, PTEN was identified to be localized in the nucleus^82,83^ and regulate genomic stability and global gene expression^84,85^. Thus, deletion of PTEN is also likely to lead to chromatin decondensation and transcription activation of genes for cell growth and mitigation of depression-like behaviors.

Our results suggest that inhibiting PTEN activity can be of therapeutic benefit in treating depression. VO-Ohpic used in this study is a specific vanadium-based PTEN inhibitor^86^. It is able to antagonize the effect of PTEN over-expression, enhance the activity of PI3K/AKT signaling pathway, and alleviate myocardial cell apoptosis^87^, and it is also able to inhibit the expression of pro-inflammatory cytokines IL-1β and TNF-α and up-regulate anti-inflammatory IL-10 expression in a sudden cardiac arrest model^88^. On the other hand, the initial consideration of developing a PTEN inhibitor in potential clinical application is discouraged by concerns that long-term systemic PTEN inhibition may lead to increased cancer risk, and because of evidence that even modest reductions in PTEN expression level lead to increased frequencies of certain tumors, particularly in breast^89,90^. Conversely, beneficial effects are also reported. For example, VO-Ohpic inhibited cell viability, cell proliferation, and colony formation, and induced senescence in hepatocellular carcinoma cells^91^. We provide proof-of-principle evidence that pharmacological inhibition of PTEN is likely to be a promising approach in treating depression. In addition, our data indicate that CRS decreased the mouse body weight and inhibition of PTEN by VO-Ohpic reversed this change (Fig. 1h), raising a possibility that PTEN plays a critical role in leptin signaling pathway, regulating energy balance^92^.

## Supplementary information

Supplementary figures and legends.
