# Supplementary material for: PTEN in prefrontal cortex is essential in regulating depression-like behaviors in mice
Source: Transl Psychiatry. 2021 Mar 26;11:185. doi: 10.1038/s41398-021-01312-y (PMC7998021; doi:10.1038/s41398-021-01312-y)
Supplement: Supplementary file 1 — Supplementary figures and legends. [file 41398_2021_1312_MOESM1_ESM.docx]

**Supplementary figures and legends**

**
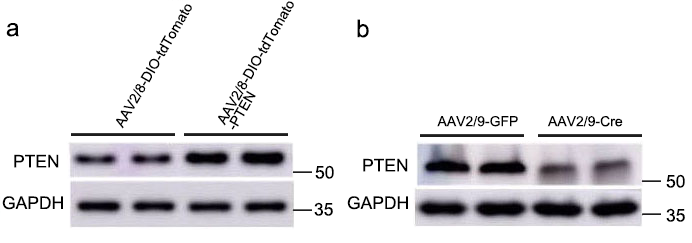
**

**Fig. S1** Verification of over-expression of *Pten* in Emx1-Cre mice, and deletion of *Pten* in *Pten*^Flox/Flox^ mice at P90. (a) Western blots showed the increase of PTEN levels in Emx1-Cre mice with injection of AAV2/8-DIO-tdTomato-PTEN compared with those with injection of AAV2/8-DIO-tdTomato. (b) Western blots showed the decreased PTEN levels in *Pten*^Flox/Flox^ mice with injection of AAV2/9-Cre compared with those with injection of AAV2/9-GFP.


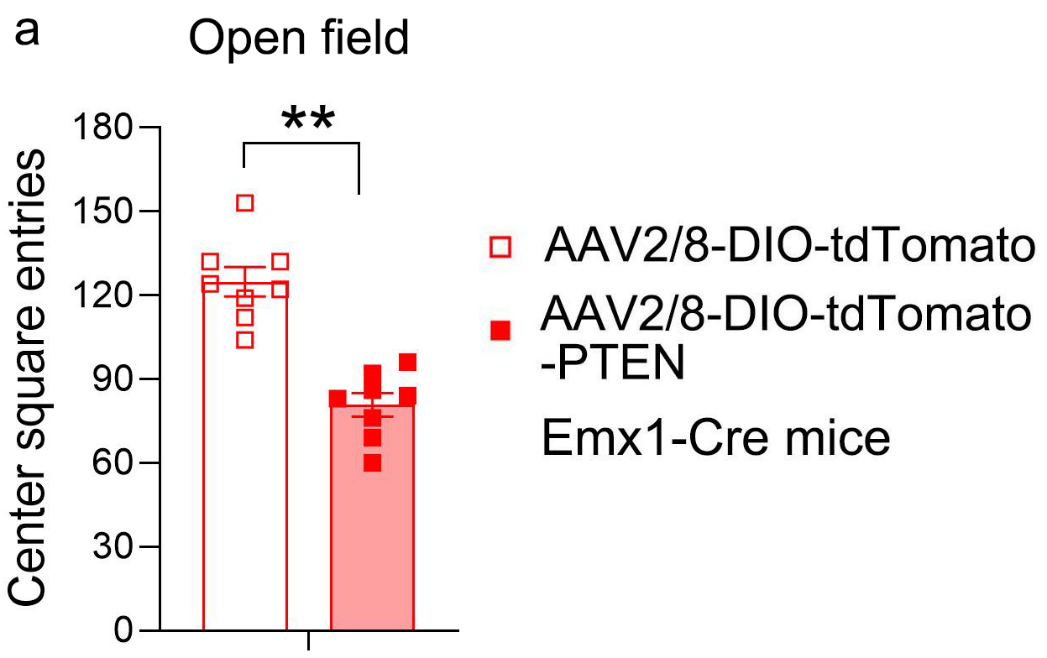


**Fig. S2** Mice with over-expression of PTEN in the PFC showed decreased entries to the center in open field test, compared with control mice. Cre-dependent over-expression of Pten was achieved in Emx1-Cre mice. n=8 in each group.


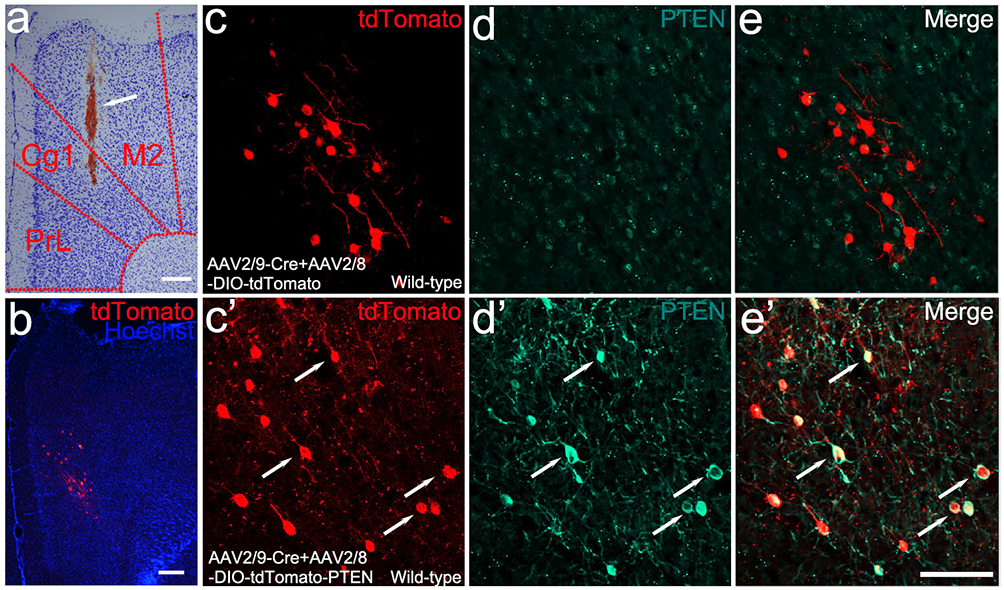


**Fig. S3** Cellular verification of PTEN expression by injection of AAV2/9-Cre and AAV2/8-DIO-tdTomato-PTEN in wild-type mice at P90. (a) The position of the needle hole for injection (arrow). (b) The extent of the virus diffusion. (c-e’) Confocal images of PTEN immunostaining (d, d’) in the PFC of wild-type mice injected with a mix of AAV2/9-Cre+AAV2/8-DIO-tdTomato (c-e) or AAV2/9-Cre+AAV2/8-DIO-tdTomato-PTEN (c’-e’). tdTomato-positive neurons (red) with intense PTEN immunoreactivity (cyan) were only observed in the PFC injected with a mix of AAV2/9-Cre+AAV2/8-DIO-tdTomato-PTEN (arrows, c’-e’). Scale bar = 200 μm (a-b) and 100 μm (c-e’). M2: secondary motor cortex; Cg1: cingulate cortex, area1; PrL: prelimbic cortex.


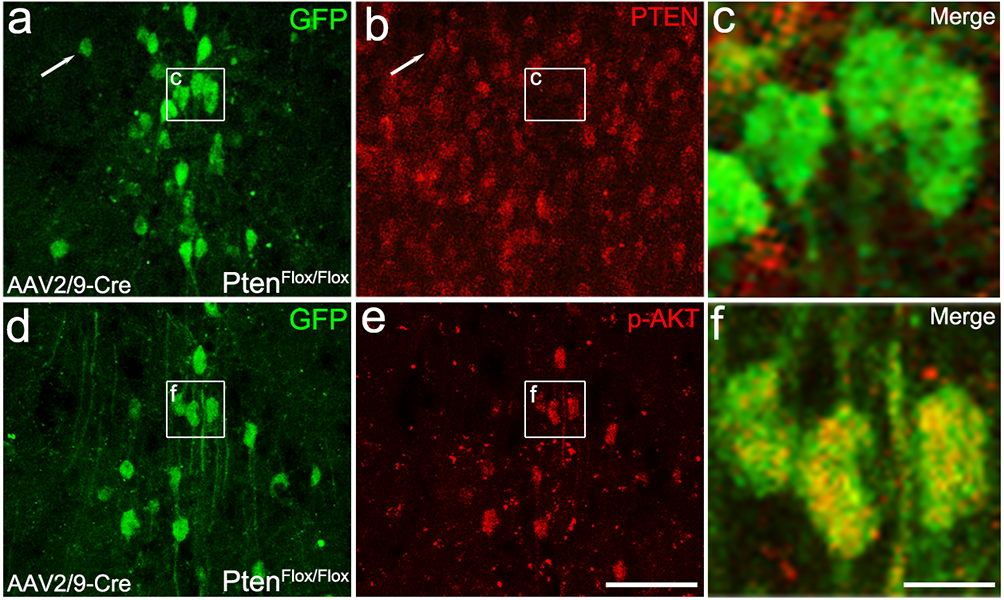


**Fig. S4** Cellular verification of PTEN deletion and increase of AKT phosphorylation in PFC of *Pten*^Flox/Flox^ mice at P90 with injection of AAV2/9-Cre. Most GFP^+^ (Cre-expressing) neurons were no longer immunostained for PTEN (red, a-c), and very few were positive for PTEN (arrows). These GFP^+^ (Cre-expressing) neurons were also positive for p-AKT (red, d-f). Boxed area in (a, b) and in (d, e) are enlarged in (c, f), respectively. Scale bar = 100 μm (a, b, d, e) and 15 μm (c, f).


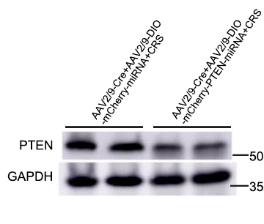


**Fig. S5** Western blots showed the decrease of PTEN levels in wild-type mice at P90 with injection of AAV2/9-DIO-mCherry-PTEN-miRNA. PTEN levels were decreased in mice with injection of AAV2/9-DIO-mCherry-PTEN-miRNA compared with those injected with AAV2/9-DIO-mCherry-miRNA.­
